# Assessing thermal performance: Dataset from an experimental study on U-value variability

**DOI:** 10.1016/j.dib.2024.111067

**Published:** 2024-10-24

**Authors:** H Alkhatib, B Norton, D Gavin, P Lemarchand

**Affiliations:** a School of Mechanical Engineering, Technological University Dublin, Ireland; b Dublin Energy Lab, Technological University Dublin, Dublin, Ireland; c Sustainability Intelligence, Technological University Dublin, Dublin, Ireland; d MaREI, the SFI Centre for Energy, Climate and Marine, Ireland; e IERC, International Energy Research Centre, Tyndall National Institute, University College Cork, Cork, Ireland

**Keywords:** U-value, Energy audit, Thermal performance, Building efficiency, Insulation

## Abstract

In order to achieve energy savings in existing buildings, there is an increasing need for energy audits and performance checks. In this regard, estimating a building's U-value is crucial, and there are a variety of methods available for achieving this. Heat flow meters can be used to measure U-value in situ. This dataset reports on an experimental campaign that evaluated the insulation and performance of the fabric elements of a building located in Dublin, Ireland. The experimental study conducted on the "Block F" building provided insights into the building's thermal performance by measuring the U-value of various windows and walls across different locations and floors. The average heat loss coefficient for windows was approximately 1.74 W/(m²K) before data cleaning and 1.99 W/(m²K) after cleaning, while walls had an average of 0.90 W/(m²K) before cleaning and 1.07 W/(m²K) after cleaning. The findings demonstrated that similar types of windows and walls components gave significantly different U-values.

Specifications Table

**Table:** 

Subject	Energy
Specific subject area	Renewable Energy, Sustainability and the Environment
Type of data	Raw, Table and Figures
Data collection	GreenTEG equipment was employed, including two U-value kits, each consisting of a heat flux data logger and two temperature sensors. Heat flow meters were placed on building elements to measure heat transfer, infrared cameras detected thermal anomalies, and data loggers recorded temperature and heat flow over time. The data logging code, record intervals, and U-value calibration factor were programmed using the GreenTEG software.
Data source location	This dataset reports on an experimental campaign that evaluated the insulation and performance of the fabric elements of a building located in Dublin, Ireland. The experimental study conducted on the "Block F" building provided insights into the building's thermal performance by measuring the U-value of various windows and walls across different locations and floors.
Data accessibility	Alkhatib, Hani; Lemarchand, Philippe; Gavin, David; Norton, Brian (2024), “A Study on U-Value Measurement for Energy Efficiency Enhancement”, Mendeley Data, V2, doi: 10.17632/xvhpnpnr3w.2 Repository name: **A Study on U-Value Measurement for Energy Efficiency Enhancement**. Data identification number: 10.17632/xvhpnpnr3w.2 Direct URL to data: https://data.mendeley.com/datasets/xvhpnpnr3w/2 Instructions for accessing these data: **Non**
Related research article	**Non applicable.**

## 1 Value of the Data

• The dataset provides detailed insights into building fabric performance, highlighting differences in U-values across various components.
• It supports the development of more accurate and reliable methods for measuring and interpreting U-values, enhancing the effectiveness of energy audits and performance evaluations.
• The dataset highlights the effect of data cleaning on U-value accuracy, showing how cleaning improves the reliability of performance assessments.
• By evaluating U-values of windows and walls, the data identifies key performance variations, offering valuable insights for building assessment and retrofit strategies.

## 2 Background

Both the wall and window elements of a facade can be engineered to (i) harness solar energy for photovoltaic electricity generation, heating, inducing ventilation and daylighting, (ii) provide varying levels of thermal insulation and (iii) store energy [[1], [2], [3]]. As a façade may need to provide each of these attributes to differing extents at particular times, achieving their optimal performance requires knowledge of the energy losses throughout the building. These, together with thermally inefficient materials and systems, lead to 40 % of global energy consumption [4,5] being used to heat, cool, light and ventilate buildings [6,7].

Previous studies have often failed to adequately address the variability in U-value readings due to different orientations and environmental conditions within the same building. This data set reports of a study aims to fill this gap by providing a comprehensive analysis of the thermal performance of various building elements across different locations and floors within the "Block F" building in Dublin, Ireland. By systematically measuring and comparing the U-values of windows and walls before and after data cleaning, this research offers new insights into the actual insulation performance and highlights the need for targeted refurbishment plans to enhance the building's energy efficiency [8,9]. The findings are particularly valuable for professionals involved in energy audits, building efficiency assessments, and thermal performance studies. The data can directly inform retrofitting decisions, helping stakeholders prioritize areas for targeted refurbishment to enhance the building's energy efficiency. Furthermore, this study provides a foundation for policy improvements aimed at achieving better building performance standards. For example, similar studies have successfully guided large-scale retrofitting projects and informed national energy-saving initiatives, showing how accurate thermal performance data can drive meaningful action.

## 3 Data Description

The dataset includes U-value measurements derived from indoor and outdoor temperature readings combined with heat flux sensor data. It covers various building orientations, floors, and multiple locations for walls and windows, with measurement durations ranging from three to fourteen days. The data cleaning process consisted of several steps to ensure accuracy and reliability. First, any jumps in the data caused by the opening of windows or doors were removed. The first 10 % of the data was excluded, and readings where the temperature difference was below 5 °C were also filtered out. Day and night data were treated separately before averaging the results. These adjustments improved the precision of the U-value calculations, providing more accurate insights into the building's thermal performance.

Table 1 presents the results obtained from the experimental campaign conducted to evaluate the insulation and performance of the fabric elements of the building in Dublin, Ireland. It details the specific locations where each test was conducted, along with the duration of each test measured in hours.

**Table 1:** Overall heat coefficient measurements in different locations of the building.

Location name	Element	Floor	Orientation	Duration of test (h)	Overall heat loss coefficient (W/(m²K))	Overall heat loss coefficient after cleaning (W/(m²K))
Wating Area*	Window	Ground	SE	95	1.08	2.1
	Wall				0.69	1.2
F014*	Window	Ground	NW	73	2.16	2.25
	Wall				1.12	1.3
F019*	Window	Ground	SW	113	2.18	2.24
	Wall				0.71	0.8
**Library (left side)**	Window	First	NE	286	1.69	Not Required
	Wall				0.86	Not Required
**Library (left side)**	Window	First	SW	385	2.31	Not Required
	Wall				0.96	Not Required
Library (right side)*	Window	First	NE	167	2.07	2.4
	Wall				0.94	1.1
**Library (right side)**	Window	First	SW	551	0.99	Not Required
	Wall				0.75	Not Required
**Hall**	Window	Second	NE	480	1.36	Not Required
	Wall				1.14	Not Required

⁎ Further data cleaning was done to minimize the effect of disturbances.

## 4 Experimental Design, Materials and Methods

To achieve a comprehensive understanding of the energy performance of a building, it is essential to measure and analyze the overall heat loss coefficient (U-value) across various locations and floors. For this, GreenTEG equipment was employed, including two U-value kits, each consisting of a heat flux data logger and two temperature sensors. Heat flow meters were placed on building elements to measure heat transfer, infrared cameras detected thermal anomalies, and data loggers recorded temperature and heat flow over time.

**Fig. 1:**
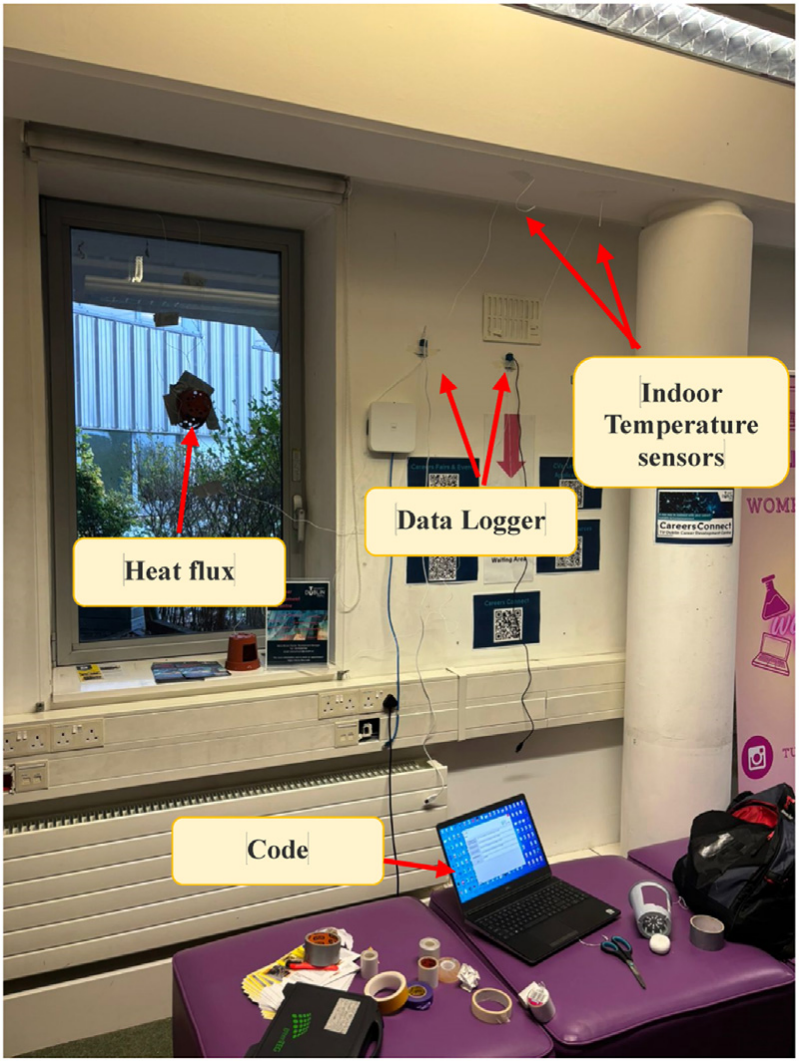
Experimental set up.

The U-value calibration process was carefully handled using the GreenTEG software. Calibration was carried out by placing the sensors in a controlled environment, and the calibration factor was adjusted based on manufacturer guidelines to ensure accuracy. Validation procedures included comparing the recorded U-values with reference materials, ensuring consistency across different locations. Equipment settings such as data logging intervals and the U-value calibration factor were determined based on the specific characteristics of the building and environmental conditions, with the goal of maximizing the reproducibility of the results. Fig. 1 shows a sample setup used in different locations, illustrating the two kits in use.

### 4.1 Equipment

Table 2 shows the accuracy, range and measuring interval of two instruments used to measure the U-value of the building fabric. Each U-value kit consists of two temperature sensors, a heat flux meter, and a data logger. These components work together to collect precise data on thermal performance. The data logger records the measurements, while a PC is used to write the code for data logging and to set the recording interval. The U-value calibration factor was also determined using specialized software. This setup ensures accurate and reliable measurement of the building's thermal properties, essential for a comprehensive energy audit.

**Table 2:** Instrument used to measure the U-value.

Measurement	Instrument		Accuracy	Range	Measurement interval
Overall heat loss coefficient		Temperature sensors	±0.5 °C	−40 to 100 °C	1 min. The kit was mounted at each fabric element for three days.
		Flux sensor	<0.11 W/m²	±300 W/m²	

## Limitations

Not applicable.

## Ethics Statement

All authors have read and follow the ethical requirements for publication in Data in Brief and confirming that the current work does not involve human subjects, animal experiments, or any data collected from social media platforms.

## CRediT authorship contribution statement

**H Alkhatib:** Conceptualization, Methodology, Investigation, Visualization, Writing – original draft, Writing – review & editing. **B Norton:** Supervision, Methodology, Investigation, Project administration. **D Gavin:** Conceptualization, Methodology. **P Lemarchand:** Conceptualization, Methodology, Supervision, Validation, Software.

## Data Availability

Mendeley DataA Study on U-Value Measurement for Energy Efficiency Enhancement (Original data)
